# Optimization of Quality, Reliability, and Warranty Policies for Micromachines under Wear Degradation

**DOI:** 10.3390/mi13111899

**Published:** 2022-11-02

**Authors:** Alexandra D. Tseni, Panagiotis Sotiropoulos, Stelios K. Georgantzinos

**Affiliations:** 1 General Department, National and Kapodistrian University of Athens, 34400 Psachna, Greece; 2 School of Science and Technology, Hellenic Open University, 26222 Patras, Greece; 3 Laboratory for Advanced Materials, Structures and Digitalization, Department of Aerospace Science and Technology, National and Kapodistrian University of Athens, 34400 Psachna, Greece

**Keywords:** MEMs, burn-in, quality, reliability, warranty, maintenance, optimization, degradation

## Abstract

This work presents an optimization technique to determine the inspection, warranty period, and preventive maintenance policies for micromachines suffering from degradation. Specifically, wear degradation is considered, which is a common failure process for many Micro-Electro-Mechanical Systems (MEMS). The proposed mathematical model examines the impact of quality control on reliability and the duration of the warranty period given by the manufacturer or the supplier to the customer. Each of the above processes creates implementation costs. All the individual costs are integrated into a single measure, which is used to build the model and derive the optimal parameters of the quality and maintenance policies. The implementation of various levels of the quality, warranty, and maintenance policies are compared with their optimum level options to highlight their contribution to the assurance and improving product quality. To the authors’ best knowledge, the introduction of a warranty period is implemented for the first time in the open literature concerning this type of optimization model for MEMs and surely can bring additional advantages to their quality promotion strategy. The proposed optimization tool provides a comprehensive simultaneous answer to the optimal selection of all the values of the design variables determining the overall maintenance and quality management approach.

## 1. Introduction

MEMS technology exhibits excessive potential for many critical applications in aerospace, automotive, medical, nuclear, and other areas. With more extensive commercialization of MEMs, many challenging manufacturing questions are into consideration including precise dimensional control and inspection, reliability testing and modeling, avoiding stiction, and maintenance strategies [1]. These productivity, quality, and reliability questions are critical issues that influence the path of MEMS to the conventional market. Therefore, MEMS manufacturers need effective tools for optimal operational decisions. These tools can be derived from the use of equivalent tools and methods developed in a traditional industry.

Preventive maintenance refers to work carried out to maintain the equipment at the desired level of operation and to avoid failures leading to production stops. In the context of preventive maintenance includes carrying out checks and inspections of equipment and the replacement of defective units. As part of preventive maintenance, the policy of periodic maintenance is applied replacement of a unit, whereby a unit is replaced at every specific operating interval [2]. The time-based replacement policy refers to the replacement of a unit at a predefined time interval, which has been derived from the manufacturer’s guidelines or existing experience. However, in many cases, the unit fails in time less than the replacement time. This case has been analyzed in [3], in which failures are divided into type I (less significant) and type II (catastrophic) failures. Type I failures require minimal replacement of a component or part while Type II failures require replacement of the entire system.

The failure-based burn-in policy is applied before the product is on the market for a short period. The tests aim to identify products that show some early failure (infant mortality) so that they can be avoided from being introduced to the market and consequently to customers. After the burn-in procedure has been applied for the specified period, the products that do not show any failure are immediately put on the market for use, while those whose component fails are replaced and then the product is put on the market [4]. On the other hand, the burn-in policy based on degradation is applied after the product has been produced and before its release and aims to identify those products in which the investigated feature exceeds a certain cut-off level without necessarily ceasing to function. The cut-off level is often lower than the failure level [5]. In these tests, products are tested under more intense conditions (elevated temperature, higher mechanical stress) so that failure or degradation occurs, which under normal conditions would occur at a much later stage of product operation. Hu et al. [6] have developed a partially observed Markov decision process to minimize the expected total burn-in cost of a product and derive some interesting structures of the optimal policy. Yu et al. [7] have developed an improved Wiener process incorporating nonlinear terms to build the degradation model of incipient fault based on the fault estimation results. For prognosis, the fast krill herd algorithm has been proposed to estimate unknown degradation model coefficients. Shafiee et al. [8] develop a mathematical model from which derives the optimal choices of maintenance policies, and the optimal time of burn-in for which the total cost is minimized.

Lifetime is defined as the time at which degradation exceeds a predefined threshold [9]. Degradation, which is caused by endogenous and exogenous factors such as environment and operating conditions, can gradually lead to failure or a product operating at an undesirable level. For this reason, it is important to model it so that predicts and avoids possible future failures. The most widely used models, which have been extensively analyzed in the literature, are the Markov models, the Gamma approach, and the Wiener process [10,11,12,13]. Of these, the Markov models are suitable for systems with discrete states while the Gamma and Wiener approximation for continuous degradation systems. Shahraki et al. [14] provide a summary of both models, highlighting the advantages of applying each of them and the limitations in their application while providing food for thought for future analysis. Li et al. [15] use the Markov model to simulate the degradation of wind turbines and derive the reliability curve. Cholette et al. [16] use the Gamma approach to derive the degradation model of a heat exchanger while from the resulting model a maintenance policy is proposed based on the condition of the heat exchanger. Zhang et al. [17] use the Wiener approach to estimate the remaining useful life and comment on its usefulness in various applications.

Age-based preventive replacement policy has been extensively studied in the literature. Jiang et al. [18] investigated an optimal age-based replacement policy, where minimal repair, corrective replacement, and imperfect repair can be carried out upon an unexpected failure with different maintenance effects. An et al. [19] studied the joint optimization of preventive maintenance and flexible job-shop rescheduling with processing speed selection, and the dynamic arrival of the new machine is considered to enhance productivity. Dong et al. [20] investigated novel reliability models and schedules optimal preventive maintenance policies, in which the closed-form reliability quantities are derived analytically and the optimum preventive replacement interval is demonstrated theoretically. Furthermore, they developed generalized reliability models [21] for multi-component systems, where each component is subject to two dependent competing failure processes, i.e., a soft failure process caused jointly by internal performance degradation and incremental damage due to effective external shock sets, and a hard failure process caused by the same random shocks. Moreover, a system may fail due to the aging of its components, or it may fail due to fatal shocks arriving from external sources. Under this mechanism of system failure, Hashemi et al. [22] proposed optimal age-based and block preventive maintenance models by considering the costs of preventive maintenance, corrective maintenance, and minimal repairs providing some formulas for the average long-run cost rate of the proposed strategies.

The issue of property degradation of mechanical systems has been extensively studied and various models have been developed to describe it. The modeling of degradation has contributed to the prediction of material behavior as well as potential failure. In addition, there are many studies related to maintenance policies for mechanical systems. In this work, however, is developed a mathematical model for finding the optimal options of various quality policies such as e.g., the maintenance interval, the burn-in method, the replacement time, and the warranty time of a key component of micromachines, which is the pin joint. The pin joint of microengines wears out the more rotations the more revolutions it performs. Thus, the optimal choices of quality policies are examined which mentioned above are considered to minimize the overall costs.

## 2. Optimization Model

Devices at the microscale can be classified into four classes [23] according to their operational interactions. Class I devices have no free moving parts, like accelerometers, pressure sensors, or strain gauges; Class II devices have moving parts with no friction or contact surfaces, such as gyros, resonators, and filters; Class III devices have moving parts with contact surfaces such as relay and valve pump; Class IV equipment has moving parts with friction and contact surfaces such as shutters, scanners, optical switches. The first three classes can achieve high reliability if adequately manufactured and packaged. For class IV devices, where frictional surfaces cannot be avoided, failure analysis and reliability assessment must be performed to drive the robust commercialization of micromachines [24].

**Figure 1:**
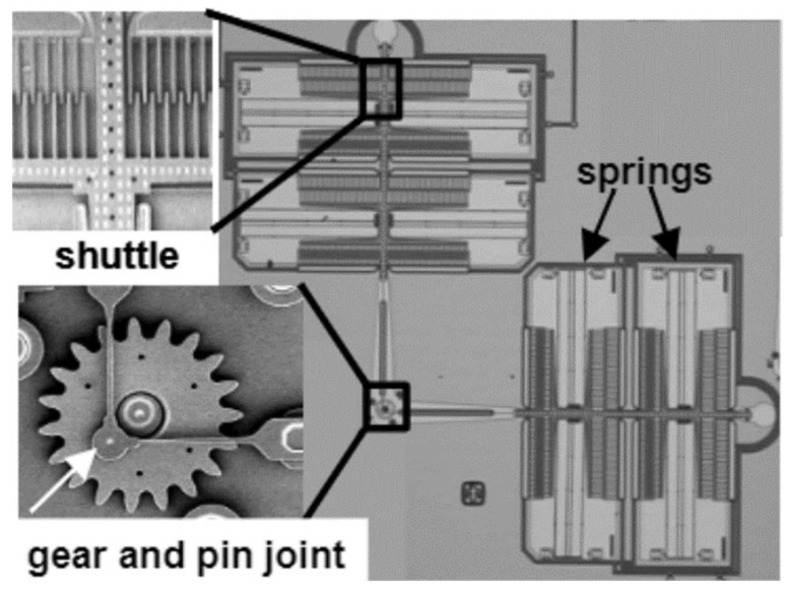
SEM image of a microengine [25,26].

The failure modes in a micromachine can be wear, friction, fracture, contamination, stiction, etc. The micromachine used in this study is the electrostatically driven microactuator (microengine) developed at Sandia National Laboratories [25] (National Technology and Engineering Solutions of Sandia, LLC., Albuquerque, New Mexico, USA). The micromachine consists of orthogonal linear comb drive actuators that are mechanically connected to a rotating gear as seen in Figure 1. By applying voltages, the linear displacement of the comb drives is transformed into circular motion. The linkage arms are connected to the gear via a pin joint. The gear rotates about a hub, which is anchored to the substrate [25]. The dominant failure mechanism was mainly identified as visible wear on the friction surface, often resulting in a seized micromotor or a micromotor with a damaged shaft seal [25,26]. Wear can be defined as the removal of material from a solid surface by mechanical action. Abrasion degradation is a very complex phenomenon, involving both the mechanical and chemical properties of the objects in contact, as well as the pressure and surface speed with which the objects are in contact.

The pin joint is a critical area for micromotors in which either less significant soft failures due to wear occur or where severe failures (hard failures) due to material breakage [27]. In the first case, the wear of the pin joint material exceeds a critical value but continues to operate while in severe failures the system stops operating. In addition, environmental conditions affect the degree of wear of the components as it has been shown that with a reduction in the humidity of the operating environment, there is an increase in the rate of wear [25,26].

Rubbing creates friction and often leads to the creation of abrasive materials or debris. The configuration of this material can lead to several different failure mechanisms. This is due to equipment-associated particle contamination, third-body abrasive particles that alter the motion tolerance, particle contamination that prevents or interferes with the movement and adhesion of surfaces, or frictional contact [28]. The wear mechanism may depend on the temperature achieved during the friction process. The material can be rubbed on the contact surface; surface materials can oxidize—Then fade, etc. Many parameters must be examined to determine the root cause of the wear, which makes analysis simple but time consuming.

**Figure 2:**
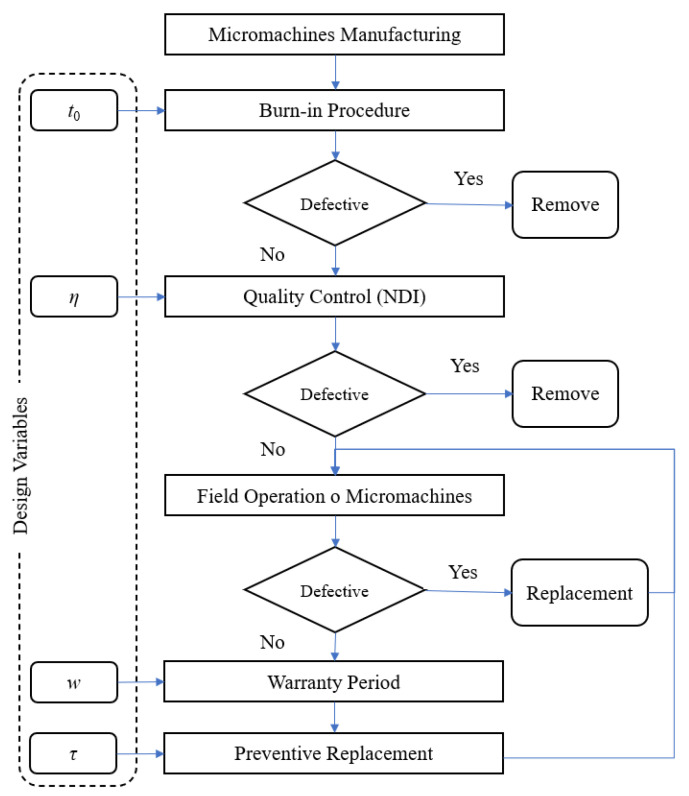
Burn-in, inspection, warranty, and preventive replacement procedures for micromachines.

To simultaneously improve quality, reliability and the warranty plan over the lifetime of micromachines, a systematic inspection, preventive replacement procedure, and warranty model have been developed, as described in Figure 2.

A post-manufacturing burn-in process for micromachines is used to identify and remove defective and early failed components. Burn-in is a crucial method for achieving reliable parts and systems, but it also exposes them to stress. For burn-in components, non-destructive inspection is applied to separate the fraction of units whose wear surpasses the specific specification threshold. The screened units, with a high level of quality, are then released for field operation until they reach the time of periodic replacement when the cost of a forthcoming failure makes it economically advantageous to replace them with new ones. The preventive replacement procedure is used to avoid failure due to the wear of standard operating units. The introduction of a warranty period can bring additional advantages to the quality promotion strategy and could therefore be adopted. For a simultaneous optimal selection of the values of the design variables determining the overall maintenance and quality management strategy, a comprehensive methodology is developed in the following sections.

### 2.1. Degradation Model

To derive the model describing the degradation of the characteristic, it is first necessary to determine the factors which influence the way affects it. The wear of the pin joint due to the rotation of the gear during the operation of a micromachine increases with the number of revolutions it performs. Therefore, the key factor in the model is the operating time, *t* [19]. The wear of the gear depends also on three other factors. These factors are the radius, *r*, of the pin, the coefficient, *c*, relating to the wear and hardness of the material, and the force, *F*, developed between the pin and the gear. This leads to a linear model in which all factors influence proportionally the wear of the pin, *X*, and the relationship is

*X*(*t*, *r*, *c*, *F*) = 2*πrcFt*. (1)

For various microengines, some of the factors are constant, while others are considered random. The radius of the pin varies for each and is considered a random factor with a mean value of *μ_r_* and a standard deviation of *σ_r_*. The force depends on the rotation frequency of the gear and consequently on the input voltage of the drive. It is therefore a random factor with mean value *μ_F_* and standard deviation *σ_F_*. Thus, at any time, for any value of radius and force, the wear *X(t)* is considered to follow the normal distribution with a mean value

(2) $$\mu_{t}=2\pi c\mu_{r}\mu_{F}t$$

and standard deviation

(3) $$\sigma_{t}=2\pi ct\sqrt{\sigma_{r}^{2}\sigma_{F}^{2}+\sigma_{r}^{2}\mu_{F}^{2}+\mu_{r}^{2}\sigma_{F}^{2}}$$

Here, we notice that more complex models considering simultaneously other failure modes could be developed. Such models may be useful for cases where the dominant failure modes have been demonstrated to be more than one.

### 2.2. Effect of Quality Control on Reliability

After the production stage of the microengines, the burn-in technique is applied to identify the defective units. Then, the non-destructive testing of all the units produced follows to remove those whose wear has exceeded the failure threshold *H*. To demonstrate the importance of quality control, it is necessary to calculate the reliability when 100% inspection of the units is applied and compare it with the reliability when none is applied.

When non-destructive testing is not applied after the burn-in process, the defective units are not detected and the reliability at any time, *t*, will be equal to the probability that the pin joint wear does not exceed the threshold failure threshold *H*

(4) $$R\left(t\right)=P\left(X\left(t\right)<H\right)=\overset{H}{\underset{0}{\int }}f_{x\left(t\right)}\left(x,t\right)dx=\Phi \left(\frac{H-\mu_{t}}{\sigma_{t}}\right).$$

When post-burn-in testing is applied then only those units whose wear is less than the failure threshold are marketed. Thus, reliability will be a bound probability and will be equal to the probability that the wear *X(t)* at any time does not exceed the failure threshold, *H*, given that it has successfully passed the burn-in procedure for a time *t_0_*. Mathematically, the reliability function will be given by

(5) $$R\left(t\right)=\frac{P\left(X\left(t\right)<H\right)}{P\left(X\left(t_{0}\right)<H\right)},$$

where $t_{0}<t<\tau ,$ and *τ* is the replacement time.

Comparing the previous equations, we observe that the reliability with the application of non-destructive testing is equal to the reliability without testing divided by a number less than unity (the probability that the post-burn-in decay is less than the failure threshold). Therefore, the application of the non-destructive control increases the reliability value.

### 2.3. Quality and Reliability

Joint optimization of quality and reliability requires the definition of the measure to be used for this purpose and the factors to be used in this model. The factors considered in finding the optimal model are the time of the burn in process, *b*, the value of the wear, *H’*, below which the system is considered to fail and the replacement time, *τ*. The measure that can be used consists of the sum of three different costs divided by the expected time of use. These costs are the quality cost, the expected failure cost, and the replacement costs.

#### 2.3.1. Quality Cost

The unit cost of quality is given by

(6) $$QC\left(\eta \right)=C_{Q}\left(\eta \right)+C_{S}\left(\eta \right)+C_{I},$$

where $C_{Q}\left(\eta \right)$ is the loss of quality, $C_{S}\left(\eta \right)$ is the cost of the rejected units, and $C_{I}$ is the cost of the inspection of each unit, which is usually fixed. The loss of quality can be calculated from Taguchi’s loss function, in which there are three different types depending on the nature of the feature under consideration. These types are the smaller the better (S-type), the larger the better (L-type), and the better the target value (T-type). In the case of the pin joint the ideal is for the wear value to be as low as possible. Therefore, the relationship that characterizes the S-Type case is chosen which is

(7) $$L\left(X\left(t_{0}\right)\right)=kX{\left(t_{0}\right)}^{2},$$

where *k* is the coefficient used to convert the deviations into economic values. The quality loss can be calculated using the expected value of $L\left(X\left(t_{0}\right)\right)$ and will be given by

(8) $$C_{Q}\left(\eta \right)=\overset{USL}{\underset{0}{\int }}L\left(x,t_{0}\right)f_{X\left(t_{0}\right)}\left(x,t_{0}\right)dx=\overset{\eta }{\underset{0}{\int }}kx^{2}f_{X\left(t_{0}\right)}\left(x,t_{0}\right)dx\;=-k\sigma_{0}\left[\mu_{0}+\eta \right]\Phi \left(\frac{\eta -\mu_{0}}{\sigma_{0}}\right)+\sigma_{0}\mu_{0}k\Phi \left(\frac{-\mu_{0}}{\sigma_{0}}\right)-k\left[\sigma_{0}^{2}+\sigma_{0}^{2}\right]\Phi \left(\frac{-\mu_{0}}{\sigma_{0}}\right)+k\left[\sigma_{0}^{2}+\sigma_{0}^{2}\right]\Phi \left(\frac{\eta -\mu_{0}}{\sigma_{0}}\right),$$

where $f_{X\left(t_{0}\right)}$ is the probability density function at the end of the burn-in application.

If a unit exceeds the failure limit, it shall either be repaired or rejected. If *q*(*η*) is the percentage of units that successfully pass the burn-in stage, then this percentage will be given by

(9) $$q\left(\eta \right)=\overset{\eta }{\underset{0}{\int }}f_{x\left(t_{0}\right)}\left(x,t_{0}\right)dx=\Phi \left(\frac{\eta -\mu_{0}}{\sigma_{0}}\right)-\Phi \left(\frac{-\mu_{0}}{\sigma_{0}}\right).$$

Moreover, if the scrap/reworked cost per unit is *s* then the scrapped portion is (1—*q*(*η*)) and the total expected scrapped cost will be

(10) $$C_{S}\left(\eta \right)=\left(1-q\right)s=\left(1-\Phi \left(\frac{\eta -\mu_{0}}{\sigma_{0}}\right)+\Phi \left(\frac{-\mu_{0}}{\sigma_{0}}\right)\right)s.$$

Inspection costs, *C_I_*, are fixed and independent of the failure threshold *η*.

#### 2.3.2. Failure Cost—Preventive Replacement Cost

The failure cost of each unit $f_{C}$ is fixed and s-independent of the time of failure and can be estimated by a one-year warranty cost or a one-time repair cost. Thus, the total failure cost depends on the reliability value at the time of replacement and is given by the relation

(11) $$FC\left(\tau \right)=f_{C}\left(1-R(\tau |t_{0}\right)).$$

In the case where we have no failure before the replacement time, the replacement based on policy will be done at that time at a replacement cost, *RC*. However, in case we have a failure before the scheduled time of replacement, then an additional replacement cost, *RC*, arises. In the more general case, the average expected failure cost will be given by the sum of the failure cost and the replacement cost. Otherwise, if it has not failed by *τ*, it should be replaced based on economic considerations, and the cost is *RC*. Thus, the expected total failure plus replacement cost at *τ* is *FC*(*τ*) + *RC.*

#### 2.3.3. Expected Time of Use

The expected time of use is a function of the burn-in time, $t_{0}$, and the replacement time, *τ*, and will be given by the following equation

(12) $$E\left[U/t_{0},\tau \right]=\overset{\tau -t_{0}}{\underset{0}{\int }}R\left(t+t_{0}|t_{0}\right)dt=\frac{1}{R\left(t_{0}\right)}\left(\tau R\left(\tau \right)-t_{0}R\left(t_{0}\right)+\overset{\tau }{\underset{t_{0}}{\int }}tf_{T}\left(t\right)dt\right),$$

where $f_{T}\left(t\right)$ is the probability density function of the failure time for a Bernstein distribution with two parameters and will be given by the equation

(13) $$f_{T}\left(t\right)=-\frac{dR\left(t\right)}{dt}=\frac{H}{\sqrt{2\pi }b}e^{\frac{{\left(H-at\right)}^{2}}{2bt^{2}}},$$

where

(14) $$a=2\pi c\mu_{r}\mu_{F},$$

and

(15) $$b=t\sqrt{{\left(2\pi c\right)}^{2}(\sigma_{r}^{2}\sigma_{F}^{2}+\sigma_{r}^{2}\mu_{F}^{2}+\mu_{r}^{2}\sigma_{F}^{2})}.$$

This is the pdf for a two-parameter Bernstein distribution.

### 2.4. Effect of the Warranty

A warranty is a contractual obligation of a manufacturer for the sale of its products. Manufacturers essentially declare that repair any damage that occurs for the duration of the warranty. But this creates an additional cost.

A product that has successfully passed the burn-in process is promoted to the market at the time, *t*_0_. Assuming that the micromachines are given a warranty period, *w*. If the product fails during its use at time *t_w_* (where *t_w_* < *w*) then under the supplier’s contract with the customer, the supplier must replace it, and this produces an additional cost *c*_2_(*t_w_*).

The expected cost for a product that comes onto the market after a burn-in period, $t_{0}$, and has a warranty period, *w*, it is given by the relation

(16) $$E\left[c_{w}\left(b\right)\right]=\overset{w}{\underset{0}{\int }}c_{2}\left(t_{w}\right)r_{b}(t_{w})dt_{w},$$

where $r_{b}(t_{w})$ is the reliability function after application of the burn-in procedure. However, if the failures occurring after the application of the burn-in process are repaired, the reliability of the product will be

(17) $$r_{b}(t_{w})=r\left(t_{0}+t_{w}\right),$$

and the expected warranty cost will be

(18) $$E\left[c_{w}\left(b\right)\right]=\overset{w+t_{0}}{\underset{t_{0}}{\int }}c_{2}\left(t-t_{0}\right)r\left(t\right)dt.$$

### 2.5. Total Cost and Optimization

The expected total system cost per unit expected usage time including warranty is described as

(19) $$TC\left(\eta ,\tau ,t_{0},w\right)=\frac{QC\left(\eta \right)+FC\left(\tau \right)+RC+E\left[c_{w}\left(b\right)\right]}{E\left[U/t_{0},\tau \right]}.$$

In fact, the upper bound of the replacement interval is usually specified and is denoted as ${Β}_{\tau }$. To minimize this cost, the optimal options of burn-in time, $t_{0}$, the replacement time, *τ*, the failure threshold, *η*, and the warranty interval, *w*, are sought. The constrained optimization model is expressed as

(20) $$\left(\eta^{*},\tau^{*},t_{0}{}^{*},w^{*}\right)=\mathrm{argmin}\left\{TC\left(\eta ,\tau ,t_{0},w\right)=\frac{QC\left(\eta \right)+FC\left(\tau \right)+RC+E\left[c_{w}\left(b\right)\right]}{E\left[U/t_{0},\tau \right]}\right\},$$

subjected to

(21) $$\eta >\mu_{0,},\;t_{0}\leq \tau \leq {Β}_{\tau }.$$

The Sequential Quadratic Programming (SPQ) method [29] is employed to solve the optimization problem. This method is a technique used to find the optimal solution to non-linear problems consisting of several subproblems. The algorithm starts from an initial hypothesis and stops after a series of iterations when the criteria set are satisfied. Calculates the optimal solution of the subproblems for which a quadratic model of the function of the original problem.

## 3. Results and Discussion

This section presents the implementation of the proposed methods using numerical examples. Firstly, selecting suitable data, the levels of the decision variables that minimize the total cost are calculated, and then the effect of the decision variables on the total cost is examined.

### 3.1. Numerical Data

To apply the model and extract the desired results and conclusions, the numerical data are presented in Table 1. It has been assumed that the surface wear of the microengines follows the normal distribution. The source of the data is given by Tanner et al. [30] considering the coefficient *c* in Equation (1), the radius of the pin joint *r*, the nominal value of the force applied between rubbing surfaces *F*, the quality loss factor, the failure per unit cost *f_c_*, the replacement cost *RC*, and the rejection cost. Here, the replacement cost within warranty *c*_2_ is also considered.

First, the values of the decision variables from which the model starts are given to calculate the cost values until the optimal solution are reached. The initial values selected are burn-in time $t_{0}$ = 200 revolutions, failure threshold *η* = 0.001 μm^3^, replacement time *τ* = 25,000 revolutions, and warranty period *w* = 10,000 revolutions. The values of the variables that minimize the total cost, which are derived from the application of the proposed model, are burn-in time $t_{0}*$ = 236 revolutions, failure threshold η* = 5.81 × 10^−3^ μm^3^, replacement time τ* = 58,945 revolutions, warranty period w* = 11,340 revolutions. The total cost for the optimal solution is *TC* = 0.0404 €/unit. It is noticed that the above values obtained from the solution of the constrained optimization model expressed in Equations (20) and (21) applying the SQP method. Here, we notice that this test case and its results are directly comparable only with the ones presented in [1]. If we apply the values of the example in [1] and we simplify the model neglecting the warranty policies to be the same as the corresponding one in [1], the obtained results are identical.

### 3.2. Comparison of Quality Policies

In order to examine the effect of quality control after the application of the burn-in process, initially in this section the different values of the total cost without quality control, i.e., without a failure limit (*η*) of the defective units, will be calculated.

**Figure 3:**
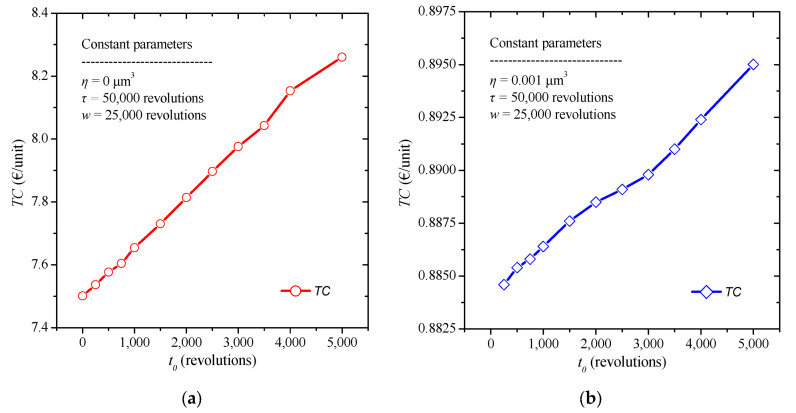
Total cost diagram for various values of burn-in time (**a**) without, and (**b**) with the presence of quality control.

Firstly, the numerical model is applied, after having reset to zero the rejection threshold *h*, changing each time the value of the time interval of the burn-in application. The constant values selected are a replacement time *τ* = 50,000 revolutions, and a warranty period *w* = 25,000 revolutions. The burn-in time $t_{0}$ varies from 0 to 5000 revolutions, while the failure threshold is *η* = 0 μm^3^, due to the absence of quality control. The results are summarized in Figure 3a. We note that the application of a longer time interval of the burn-in process reasonably increases the total costs. In addition, we find a first insight into the values of the total costs incurred in the absence of quality control.

To draw conclusions on the impact of quality control on the total cost, the proposed model is applied by considering the quality control expressed through the failure threshold. A failure threshold *η* = 0.001 μm^3^ is chosen to calculate the total cost. The constant values selected are a replacement time *τ* = 50,000 revolutions, and a warranty period *w* = 25,000 revolutions. The burn-in time *t*_0_ varies from 0 to 5000 revolutions, while the failure threshold is *η* = 0.001 μm^3^, due to the quality control process. The results are summarized in Figure 3b. From the above results we notice that the total cost increases with the increase of the burn-in time. However, we observe that while doubling the time of the burn-in process there is no meaningful change in the value of total cost. Specifically, by increasing by 1000 revolutions the burn-in time the total cost increases less than 5% in all cases. Moreover, we observe that, in this case, the absence of quality control can produce ten times (1000%) greater total cost than the one with quality control in most cases.

**Figure 4:**
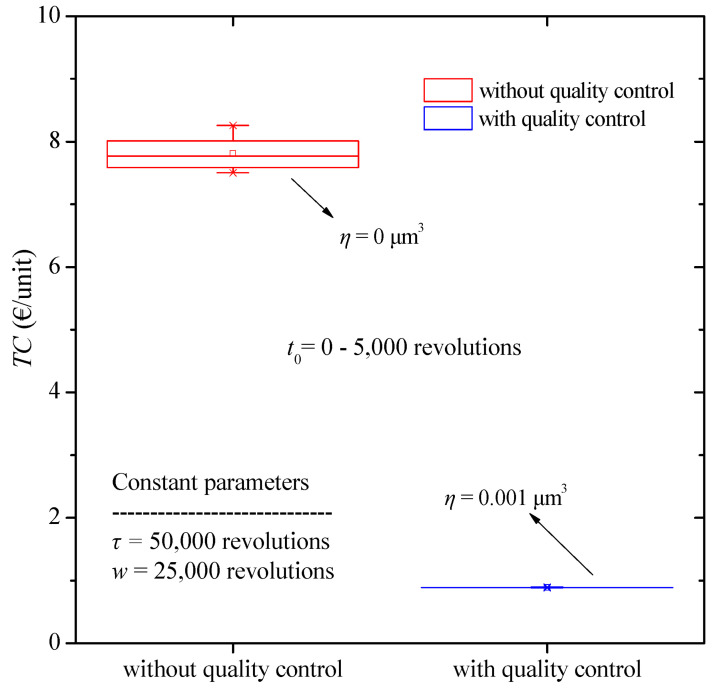
Total cost boxplot diagram for various values of burn-in time with and without quality control.

For a more detailed analysis of the results and to allow comparison of the results, the box plots for total costs as a function of the change in burn-in time when quality control is or is not applied are presented in Figure 4. We observe a sharp decrease in the cost values with application of quality control. Furthermore, the results obtained without quality control show a considerably larger dispersion.

**Figure 5:**
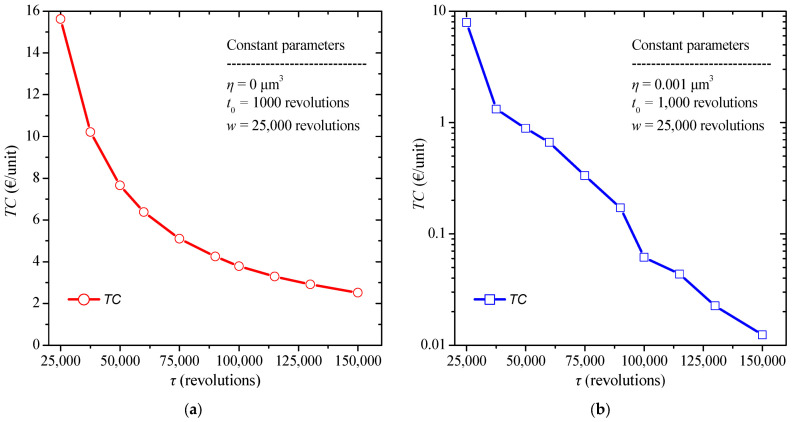
Total cost diagram for various values of replacement time (**a**) without, and (**b**) with the presence of quality control.

The same procedure is then repeated keeping all variables constant except for the replacement time interval, *τ*. The constant values selected are a burn-in time $t_{0}$ = 1000 revolutions, and a warranty period *w* = 25,000 revolutions. The replacement time, *τ*, varies from 25,000 to 150,000 revolutions, while the failure threshold is *η* = 0 μm^3^, due to the absence of quality control. The results are summarized in Figure 5a. It is obvious that increasing the replacement interval leads the reduction of overall costs when no quality control is applied.

A similar procedure is repeated keeping all the factors of the model constant, each time changing the replacement time applying quality control. The constant values selected are a burn-in time $t_{0}=$ 1000 revolutions, a warranty period *w* = 25,000 revolutions, and a failure threshold *η* = 0.001 μm^3^. The replacement time varies again from 25,000 to 150,000 revolutions. The results are illustrated in Figure 5b. We can observe that with the increase of the replacement time the total cost as the replacement cost decreases. Furthermore, increasing the replacement time, it contributes significantly to reducing costs. Near at the optimal value of 58,945 rotations the total cost is about 0.85 €/item, while when the value is under sampled the total cost increases and approaches 7.5 €/item. This is because the sooner a component is replaced the less likely it will fail. These results suggest that increasing the preventive replacement time has a significant effect on the reduction of the total cost. The rate of reduction decreases with increasing replacement time.

**Figure 6:**
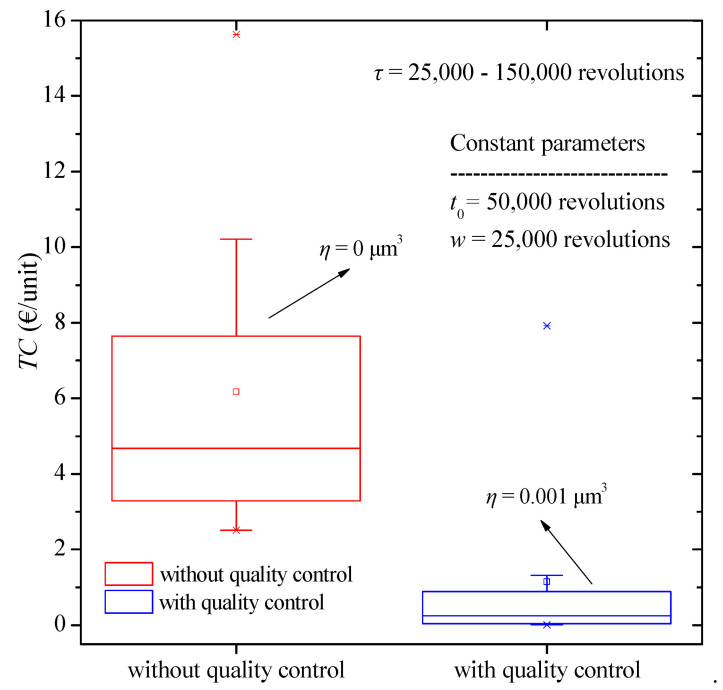
Total cost boxplot diagram for various values of burn-in time with and without quality control.

The boxplot diagram in Figure 6 is then assembled from the results of previous analysis for the various values of the replacement time. In this case as well, we observe a reduction in total cost when quality control is applied as well as a smaller dispersion of the results appears. Moreover, we observe that, in this case, the absence of quality control can produce 100 times greater total cost than the one with quality control in some cases.

**Figure 7:**
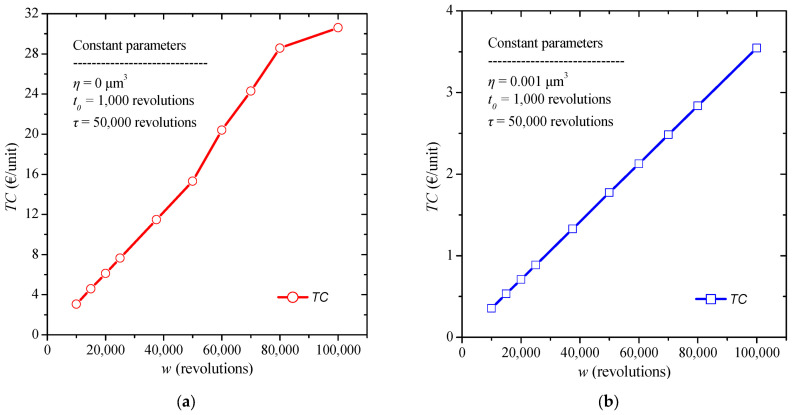
Total cost diagram for various values of warranty period provided by the manufacturer (**a**) without, and (**b**) with the presence of quality control.

Furthermore, the application of the model without quality control is implemented by varying this time the length of the warranty period provided by the manufacturer. The constant values selected are a burn-in time $t_{0}$ = 1000 revolutions, and a replacement time *τ* = 50,000 revolutions. The warranty period, *w*, varies from 10,000 to 100,000 revolutions, while the failure threshold is *η* = 0 μm^3^, due to the absence of quality control. The results obtained are shown in Figure 7a. Ιt can be observed that the total cost increases with the increase in the total warranty period provided by the manufacturer.

The warranty period is also examined for its influence on total costs when quality control is applied. The constant values selected are a replacement time *τ* = 50,000 revolutions, and a burn-in period of *t*_0_ = 1000 revolutions. The warranty time w varies from 10,000 to 100,000 revolutions, while the failure threshold is *η* = 0.001 μm^3^, due to the quality control process. The results are summarized in Figure 7b. From the presented results we additionally conclude that with an increase in the warranty period the total cost increases as more failures occur during this period which must be replaced by the manufacturer.

**Figure 8:**
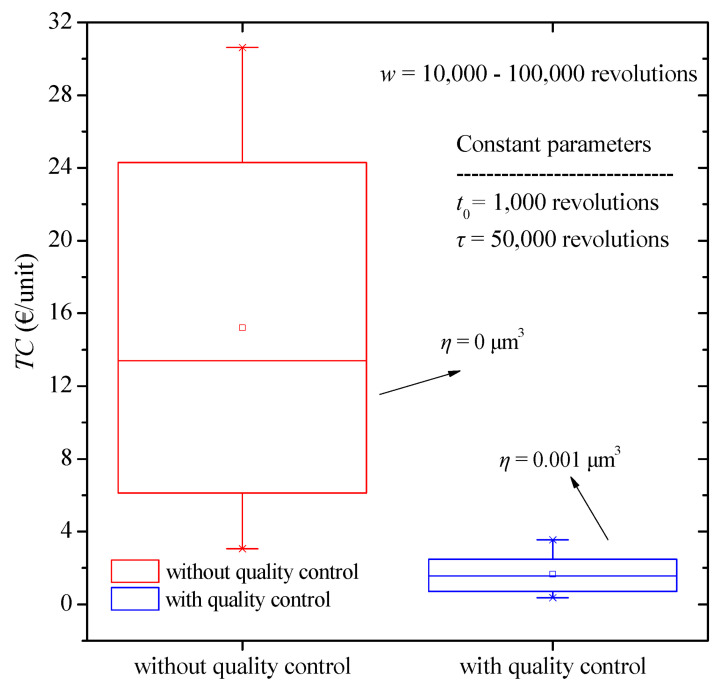
Total cost boxplot diagram for various values of warranty period with and without quality control.

To further compare the effect of period of the warranty on the total cost, a corresponding boxplot is presented in Figure 8. And in this case, we come to the same conclusion, i.e., the noticeable reduction in total costs and in the less dispersion of results by applying quality control.

**Figure 9:**
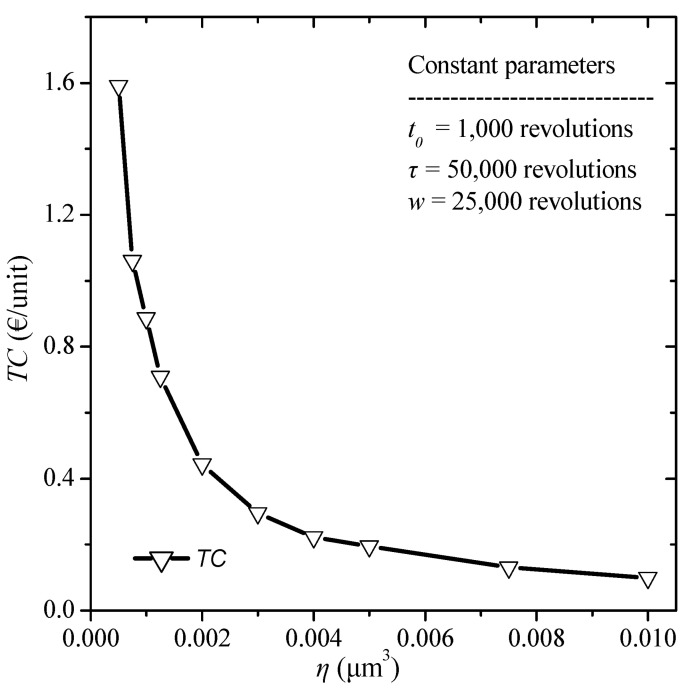
Total cost diagram for various values of failure threshold.

The effect of the quality control threshold, i.e., the different values of the failure threshold, on the total cost is finally examined. The constant values selected are a replacement time *τ* = 50,000 revolutions, a burn-in time $t_{0}=$ 1000 revolutions, and a warranty period *w* = 25,000 revolutions. The failure threshold varies from 0.0005 to 0.0100 μm^3^. The results are depicted in Figure 9.

From the above results we observe that as the failure threshold decreases the total cost increases, which is since the lower the failure threshold, the more units are rejected after the burn-in stage and are considered to fail.

An important observation concerns the dispersion of results. We observe that when there is no quality control the results show a large dispersion from the mean value and a large range from the minimum and maximum values, while when there is a quality control for all three decision variables it is observed that the results are clustered around the mean value. We also observe that increasing the time of application of the burn-in procedure only slightly increases the total cost with values close to 0.885 €/unit, since its application for a longer period contributes to the detection of more defective units which are not passed on to the consumer.

## 4. Conclusions

In this paper, a mathematical model with economic cost factors was developed based on the degradation of an important component of micromotors, the pin. This model could be applied to other components of micromotors, whether electrical or mechanical. The wear of micromotors increases with the number of revolutions of the pin and depends on three other factors, namely the radius of the pin, the hardness coefficient of the material and the force developed between the gear and the pin. Based on the calculation of the degradation relationship, which is exhibited by the micromotors and the rotation pin which is the contact point between the actuator and the gear, the various costs were calculated. Quality costs, failure costs, replacement costs and warranty costs were considered. Deriving the total cost expression, the values of the decision parameters that minimize the total cost were calculated using computational techniques.

Using a parametric analysis to study the sensitivity of the solution on the decision variables, the following conclusions are arisen:

- the presence of quality control significantly reduces the total costs for all the decision variables. Concerning the numerical example, the total cost is reduced more than ten times (1000%) when quality control is applied in most cases.
- a large dispersion from the mean with a large range from the minimum and maximum values exists without a quality control, while when a quality control is implemented, the results are clustered around the mean for all the decision variables.
- the increase in the burn-in time slightly increases the overall cost, while its application for longer time contributes to the detection of more defective units which are not passed on to the consumer. Concerning the numerical example, increasing by 1000 revolutions the burn-in time the total cost increases by less than 5% in all cases.
- the increase in the replacement interval is observed to contribute significantly to the reduction of total cost since the sooner a component is replaced, the less likely it is to fail.
- the increase in the warranty period provided by the manufacturer increases the total cost significantly.

## Figures and Tables

**Table 1:** Numerical data applied to the proposed models.

*c* (μm^2^/N)	*r* (μm)	*F* (N)	s.d. of *r* (μm)	s.d. of *F* (N)	Quality Loss Factor	$f_{C}$ (€/Unit)	*RC* (€/Unit)	Rejection Cost (€/unit)	*c*_2_ (€/Unit)
0.0003	1.5	3 × 10^−6^	0.075	1.5 × 10^−7^	10^10^	1000	50	50	70
